# Plasma oxytocin changes and anti-obsessive response during serotonin reuptake inhibitor treatment: a placebo controlled study

**DOI:** 10.1186/1471-244X-13-344

**Published:** 2013-12-23

**Authors:** Mats B Humble, Kerstin Uvnäs-Moberg, Ingemar Engström, Susanne Bejerot

**Affiliations:** 1 Psychiatric Research Centre, Örebro County Council, School of Health and Medical Sciences, Örebro University, Örebro, Sweden; 2 Department of Animal Environment and Health, Swedish University of Agricultural Sciences, Skara, Sweden; 3 Department of clinical neuroscience, Karolinska Institutet, Stockholm, Sweden; 4 Psychiatric Research Centre, Box 1613, Örebro SE-701 16, Sweden

**Keywords:** Obsessive-compulsive disorder, Oxytocin/plasma, Serotonin, Serotonin uptake inhibitors, Treatment response, Randomized controlled trial, Autism spectrum disorder, Placebo response

## Abstract

**Background:**

The drug treatments of choice for obsessive-compulsive disorder (OCD) are serotonin reuptake inhibitors (SRIs). However, a correlation between the neuropeptide oxytocin in cerebrospinal fluid and the severity of OCD has previously been shown, and oxytocin and serotonin are interconnected within the brain. Few studies have investigated whether SRIs have any effect on oxytocin; thus, our aim was to explore the possibility that oxytocinergic mechanisms contribute to the anti-obsessive effect of SRIs.

**Method:**

In a randomized, double-blind trial, comparing SRIs (clomipramine and paroxetine) with placebo in 36 adults with OCD (characterized for subtypes), plasma oxytocin was measured with radioimmunoassay after plasma extraction, at baseline, after 1 week, and after 4 weeks of treatment, and related to baseline severity and clinical response after 12 weeks, as measured by the Yale-Brown Obsessive Compulsive Scale (Y-BOCS).

**Results:**

Baseline oxytocin levels correlated positively with baseline Y-BOCS ratings, but only among the future SRI responders. Patients with early onset of OCD had higher baseline oxytocin. During treatment, plasma oxytocin did not differ between SRI and placebo treatment. In SRI responders, plasma oxytocin first decreased and then increased; in non-responders (to SRI as well as to placebo), the reverse was the case. After 4 weeks, treatment responders had attained higher oxytocin levels compared to non-responders. The intra-individual range (i.e. the variability) of plasma oxytocin between measurements was the measure that best differentiated responders from non-responders. This range was higher in responders than non-responders, and lower in patients with autistic traits.

**Conclusions:**

SRIs have highly variable effects on plasma oxytocin between individuals. The associations between baseline oxytocin and OCD severity and between oxytocin changes and treatment response support the notions that oxytocin is involved in OCD pathophysiology, and that the anti-obsessive effects of SRIs are partly exerted through oxytocinergic mechanisms.

## Background

Obsessive-compulsive disorder (OCD) is a fairly common psychiatric disorder with variable severity [1]. The course is often chronic, and OCD is commonly comorbid with depression [2]. Furthermore, the clinical picture of OCD is heterogeneous and various subtypes characterized by concurrent tics, autistic traits, poor insight, and obsessive-compulsive personality disorder have been proposed [3-8]. At an early stage, the initial findings that clomipramine has a specific anti-obsessive effect directed the focus of neurochemical hypotheses on OCD towards the monoamine serotonin [9-12]. Subsequent research on the psychopharmacology of OCD has to a great extent been determined by the well-documented effect of serotonin reuptake inhibitors (SRIs, e.g. clomipramine and the selective serotonin reuptake inhibitors (SSRI)), leaving the serotonergic hypothesis of OCD as the most influential [13,14]. However, up to 50% of OCD patients will not or only partially respond to SRIs [13,15], and, even if indications of dopamine and glutamate involvement are acknowledged, no consensus exists whether other effects, downstream of the SRI-induced changes of serotonin availability, are involved in the clinical benefit [16,17].

The nonapeptide oxytocin is one of the candidates, possibly involved in obsessive-compulsive neurochemical pathways [16,18,19]. Oxytocin is released from hypothalamic neurons in the paraventricular (PVN) and supraoptic (SON) nuclei via axon terminals in the posterior pituitary as a hormone into the peripheral plasma pool. In addition, the same magnocellular neurons of these nuclei exert somatodendritic volume transmission of oxytocin, contributing to diffuse and long-lasting effects in adjacent brain areas [20,21] and also to the oxytocin content of the cerebrospinal fluid (CSF). More recently, evidence for immediate, targeted, “wired” transmission of oxytocin has been presented; axonal projections, mainly from PVN, connect directly with the central nucleus of amygdala, nucleus accumbens, ventral hippocampus, and other brain areas where oxytocin receptors are present [22-24]. Peripherally, there is paracrine/autocrine synthesis of oxytocin in the female and male genital tracts, the pancreas and the heart [25,26], but their contribution to plasma levels is unknown.

When it comes to cerebral functions, research indicates that oxytocin promotes innate mechanisms for maternal, affiliative, pro-social, sexual/reproductive and aggressive behaviors and social recognition [27-29]; a deficiency of some of these functions being relevant for autism spectrum disorders (ASD) [29,30]. On the other hand, increased oxytocin induced behaviors and cognitions, related to maternal, affiliative, grooming [19,31] and sexual functions, may appear similar to several core symptoms of OCD. Consequently, Leckman et al. hypothesized that some forms of OCD may represent an over-expression of such oxytocin related innate mechanisms [18]. However, as OCD and ASD often co-occur [4-6,32-34] the opposite directions of their purported oxytocinergic derangements may seem paradoxical.

Treatment with intranasal oxytocin was reported to improve OCD in an early case report [35], but two negative randomized, placebo-controlled trials refute this finding [36,37]. Moreover, it is unclear how intranasal administration of oxytocin affects the oxytocinergic transmission in relevant parts of the brain [38,39]. CSF levels of oxytocin have been measured in OCD patients, also with inconsistent results. In the study by Swedo et al. [40], CSF-oxytocin of 43 children/adolescents correlated positively with depression, but not with OCD symptom severity. In the next study [41], CSF-oxytocin was elevated compared to controls in 22 OCD adult subjects without history of tic disorders, and in these patients CSF-oxytocin was also positively related to OCD severity, as measured by the Yale-Brown Obsessive Compulsive Scale (Y-BOCS). This finding supported the above mentioned oxytocinergic OCD hypothesis [18], but a subsequent study [42] found no CSF-oxytocin difference between OCD and control cases and no relation to Y-BOCS ratings, however, only 14 patients with OCD were included. More recently, an animal model was reported [19], supporting that oxytocin gives rise to grooming compulsions through links between the PVN and the central nucleus of amygdala.

Several mechanisms connecting serotonin and oxytocin in the brain have been reported [43-46], and oxytocin has even been suggested to mediate the therapeutic effects of SSRIs [47,48]. Serotonin 1A receptors (5-HT_1A_) are probably the main mediators of the effect of serotonin on oxytocin neurons (at least acutely), but also 5-HT_2C_ and 5-HT_4_ seem to be of importance [43,49,50]. In OCD, long-term SSRI treatment has been shown to down-regulate 5-HT_1A_ receptors [51], likely to decrease hypothalamic oxytocin out-put [44]. However, it has been suggested that the clinical benefit in OCD results from a down-regulation of presynaptic 5-HT_1D_ receptors in the orbitofrontal cortex, leading to increased transmission over postsynaptic 5-HT_2A_ receptors [14]. Concerning serotonin and peripheral oxytocin, plasma oxytocin in rats increased within one hour of administration of the SSRIs citalopram or zimelidine [47], while it was unchanged when oxytocin was assessed after 10 days of fluoxetine administration [52], suggesting that timing is important when assessing the effects of antidepressants on plasma oxytocin.

To our knowledge, only three previous studies have investigated oxytocin changes during SRI treatment in humans. In the first of these [53] 16 children/adolescents with OCD were studied. Clomipramine treatment, ranging between 8.5 and 34 months, caused an overall increase of CSF oxytocin by 11%. Intriguingly, however, the individual clinical response was negatively correlated to CSF oxytocin changes, i.e. those with the least increase of CSF-oxytocin were the most improved. Since this study only included treatment responders and no placebo group, conclusions on the pharmacological effects of SRIs on the oxytocin system should be viewed with caution. In the next study [54], plasma oxytocin was measured in 40 patients with major depression before and after successful treatment, which was SRIs (venlafaxine or SSRI) in 19 cases. When compared to a control group, the patients had significantly lower plasma oxytocin at baseline, however, no difference between pre-treatment and post-treatment oxytocin levels was found. All included patients were treatment responders, and the time span between samples was not conveyed. Recently, a third study [55] reported on plasma oxytocin at baseline and after 12 weeks’ SSRI treatment in 16 adult patients that were successfully treated for major depressive disorder. No difference was found. Consequently, in none of these three studies was placebo-treated patients used as control, nor were responders compared to non-responders. Two of them dealt with depression and only one [55] applied a fixed time interval for the second oxytocin sample.

In summary, we still do not know whether oxytocin is critically involved in OCD pathogenesis or not, and if so, whether the oxytocinergic activity should be increased, decreased, or changed in other ways in order to improve the clinical state. Furthermore, the effects of SRI treatment on the human oxytocinergic system are still poorly characterized, since no study has explored differences between antidepressants and placebo, or temporal changes during the early phases of treatment.

### Aims

We wanted to explore whether pre-treatment plasma oxytocin is related to OCD severity and other clinical features in adult patients with OCD, as compared to previous studies of CSF oxytocin. Furthermore, we planned to investigate in a placebo controlled trial whether SRI treatment in humans are linked to changes of plasma oxytocin and, if so, the direction and magnitude of these changes. Finally, we aimed at testing the hypothesis that oxytocin changes correlate with and possibly predict anti-obsessive response, by following the temporal pattern of plasma oxytocin during the first four weeks of SRI treatment.

## Methods

### Study design

In a multicenter drug trial, carried out 1992–1993, comparing paroxetine with clomipramine and placebo for the treatment of OCD [56], our center included some biochemical measurements in addition to the blood samples for safety measures. The blood samples were taken at baseline (before any drug treatment), after 1 week and after 4 weeks of drug treatment. This schedule was chosen in order to detect early changes of biochemical measures that could possibly predict the clinical response after 12 week’s treatment. The blood samples were analyzed in 1995.

### Patient population

Details on the inclusion of 36 patients in this biochemical extension of the drug trial have been published elsewhere [57]. Briefly, patients could be included if they fulfilled DSM-III-R criteria for OCD (the DSM-III-R and DSM–IV criteria for OCD are widely viewed as interchangeable [58]) with at least 6 months’ duration and consented to the blood sampling. Customary exclusion criteria were applied. At the time of inclusion, no patient had been taking antidepressant agents of any kind during the preceding three months, and two thirds (n = 24) were SRI treatment naïve. Females in reproductive age were ascertained not to be pregnant and informed to use effective contraceptive methods, if applicable. For demographic data, see Table 1.

**Table 1:** Demographics and clinical response according to randomized treatment

	**All patients n = 36**	**Randomized treatment**			
		**Paroxetine n = 18**	**Clomipramine n = 9**	**Placebo n = 9**	**Statistical significance**
Sex, males/females, n (% males)	17/19 (47)	8/10 (44)	7/2 (78)	2/7 (22)	ns
Age, years	40.7 ± 12.7	38.7 ± 11.8	41.0 ± 13.5	44.6 ± 14.3	ns
OCD, age of onset, years	14.5 ± 6.5	15.2 ± 5.1	13.3 ± 8.7	14.2 ± 7.3	ns
OCD duration, years	26.2 ± 14.8	23.4 ± 13.7	27.6 ± 15.3	30.3 ± 16.8	ns
Y-BOCS, baseline	25.3 ± 5.9	24.6 ± 5.7	24.8 ± 6.8	27.2 ± 5.7	ns
Y-BOCS,% decrease, LOCF	34 ± 27	41 ± 31	38 ± 20	17 ± 21	ns
Y-BOCS,% decrease, completers^a^	44 ± 26 n = 24	51 ± 24 n = 14	56 ± 10 n = 4	22 ± 25 n = 6	F: 4.1 (2, 21)*
MADRS, baseline	12.2 ± 7.8	10.2 ± 6.4	8.4 ± 3.6	19.9 ± 8.7	F: 8.6 (2, 33)***
MADRS, endpoint, LOCF	8.3 ± 6.8	5.8 ± 5.6	9.3 ± 8.1	12.1 ± 6.2	ns
Premature discontinuation, n (%)^b^	12 (33)	4 (22) (4, 8, 8, 10 w)	5 (56) (1, 2, 8, 10, 10 w)	3 (33) (10, 10, 10 w)	ns
PGE, 1 or 2 at endpoint, n (%)	18 (50)	11 (61)	5 (56)	2 (22)	ns
Responders, n (%)	17 (47)	11 (61)	5 (56)	1 (11)	χ^2^: 6.4 (2)*

Numbers are mean values ± standard deviations, unless otherwise specified.

Statistics for comparisons between groups: F = one-way ANOVA statistics for continuous measures; *χ*^2^ = Pearson’s chi-squared test for categorical measures.

LOCF = endpoint data, calculated from 12 weeks’ ratings or last observation carried forward; MADRS = Montgomery Åsberg Depression Rating Scale; OCD = obsessive-compulsive disorder; PGE = Patient’s Global Evaluation; Y-BOCS = Yale-Brown Obsessive Compulsive Scale; Y-BOCS% decrease = endpoint Y-BOCS score related to Y-BOCS at baseline.

^a^Completers continued their randomized treatment for 12 weeks.

^b^Numbers within second parentheses represent the number of weeks of double-blind treatment for each individual discontinuer.

*p < 0.05; **p < 0.01; ***p < 0.001; ns = non-significant.

### Ethics

The study was approved by the Research Ethical Committee of the Karolinska Institutet, Stockholm, Sweden, and informed consent was obtained from all participants.

### Drug administration

After randomization (2:1:1 ratio), double-blind drug treatment was given for 12 weeks with increasing, flexible doses of either paroxetine 20–60 mg/day (n = 18), clomipramine 50–250 mg/day (n = 9) or placebo (n = 9). Zopiclone 7.5 mg h.s. for insomnia was permitted, if necessary, but cognitive or behavioral psychotherapy was not allowed during the study. For clinical results, see Table 1 and [57].

### Assessment instruments

Clinical data including OCD history, severity and subtype were recorded in a standardized, semi-structured way. The following rating instruments were used: Y-BOCS [59], in order to quantify OCD symptom severity and to evaluate treatment response; National Institute of Mental Health Global Obsessive Compulsive Scale (NIMH-GOCS) [60], a complementary method to measure global severity of OCD; Montgomery Åsberg Depression Rating Scale (MADRS) [61], a clinician rated instrument for measuring depressive symptoms, and the Patients’ Global Evaluation (PGE), a self-rating version of the 7 graded Clinical Global Impression-Improvement scale [62]. Poor insight was evaluated clinically, and autistic traits were assessed with assistance of the High-functioning Autism/Asperger syndrome Global Scale (HAGS) [33]. It covers functional impairment, social and emotional reciprocity, social competence, interests, rigidity, values, self-reflection, speech and language, body posture, gestures, facial expression, and eye contact. The rating consists of four different levels: 1 = an exceptionally empathic and socially competent personality; 2 = more or less normal, “like most people”; 3 = an emotionally blunt and pathological personality with autistic traits, clearly noticeable during the interview; and 4 = an extremely odd personality; the person gives a peculiar, and clearly autistic, impression early in the interview. A HAGS score of 3 or more was considered as sign of autistic traits. Eleven patients were assessed as having autistic traits. To our knowledge, at least five of these patients were later formally diagnosed with ASD.

### Response criteria

Treatment response was defined as at least 25% decrease of scores on the Y-BOCS in conjunction with a rating of 1 or 2 (“very much improved” or “much improved”, respectively) on the PGE at endpoint. For premature discontinuers, the last observations were carried forward. PGE, rather than the clinician’s rating of global improvement was chosen in order to also take the patient’s opinion into account. Applying these criteria, 17 of the 36 patients (59% of SRI treated patients) responded to treatment.

### Oxytocin analysis

Blood samples were obtained by cubital venepuncture, between 8 h00 and 9 h00 a.m., when patients had been fasting from midnight and before the morning dose of medication was taken. The sampling was performed by one of two nurses, known to the patients, on each occasion in the same quiet room, at normal room temperature and under comfortable circumstances. Samples were taken at baseline, after 1 week’s double-blind treatment and after 4 weeks of treatment. The samples were collected in tubes containing heparin (10 IU/mL) and Trasylol (500 IU/mL) and centrifuged. Plasma was separated, frozen at -70°C, and blindly analyzed in the same assay in 1995. The concentration of oxytocin was measured with a specific radioimmunoassay (RIA) described by Stock and Uvnäs-Moberg [63]. Briefly, plasma samples were extracted on SEP-PAK C_18_ cartridges prior to assay. The recovery of this extraction procedure was 95.3 ± 10.1%. For the assay, antiserum K19 (Milab, Malmö, Sweden) was used, which has a cross-reactivity at 70% relative binding (B/BQ) of 0.01% with arginine(A)-vasopressin, <0.01% with lysine(L)-vasopressin and 0.1% with A-vasotocin. The limit of detection is 2 fmol/ml and the intra- and inter-assay coefficients of variation are 11.2 and 13.0% respectively.

### Data analysis

Based on previous literature, no prediction of the expected direction of findings was possible. The patients treated with paroxetine and clomipramine were initially analyzed separately, but when they were compared on relevant parameters no meaningful differences between these two groups were found. As the clomipramine cases, when adjoined, did not change the results of the paroxetine cases, these two treatment groups were merged to the SRI group, having the same putative mechanism of action [13,14]. Since most oxytocin measurements were non-normally distributed, all oxytocin values are reported as medians with 1^st^ and 3^rd^ quartiles in parentheses, and nonparametric tests (Mann–Whitney U-test (MW) and Spearman Rank Order Correlation) were utilized. For other group comparisons one-way ANOVA or Chi-2 statistics were used. ANOVA of repeated measures of the oxytocin changes turned out invalid due to the non-normal distributions. This and low sample size prevented multivariate methods to be used. Thus, nonparametric tests of the measures and of the differences between the repeated measures are reported. Also, we calculated the intra-individual range (the difference between the maximal and minimal plasma oxytocin levels among each patient’s three (n = 32) or two (n = 4) samples over the 4 weeks), intended to be a measure of flexibility or responsivity of the oxytocinergic system. The data on age of onset were categorized into tertiles, which corresponded to childhood, adolescence and adult onset, respectively. Statistica 64, version 10, StatSoft Inc. was used. Probabilities < 0.05 were assumed as significant and, when relevant, Bonferroni’s adjustment for multiple comparisons was judiciously implemented. However, due to the explorative nature of this study, also the non-adjusted results are presented.

## Results

### Baseline oxytocin and clinical features of OCD

**Figure 1:**
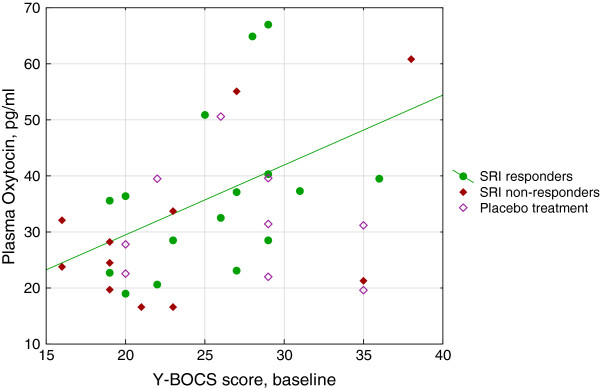
**Correlation between baseline severity of OCD and baseline plasma oxytocin according to SRI response.** Severity of OCD is indicated by total score on the Y-BOCS. Due to non-normal distribution of oxytocin, non-parametric analyses were performed; in the figure, however, a regression line for the SRI responders derived from a Pearson’s correlation (y = 4,5445 + 1,2468*x; r = 0,42; p = 0,10) is included. Spearman’s correlation statistics for the various groups: The entire sample: rho = 0.35, n = 36, p = 0.037. SRI responders: rho = 0.58, n = 16, p = 0.019. SRI non-responders: rho = 0.24, n = 11, p = 0.47. Placebo treated: rho = -0.18, n = 9, p = 0.64. OCD = obsessive-compulsive disorder; SRI = serotonin reuptake inhibitor; Y-BOCS = Yale-Brown Obsessive Compulsive Scale.

Plasma oxytocin at baseline was non-normally distributed and had a median of 31.3 (22.7, 39.5) pg/ml and a mean of 33.4 (±13.5). The distribution appeared bi- or trimodal, with a distinct high mode (50–67 pg/ml) and two less distinct lower modes (16–25 and 27–41 pg/ml, respectively); see Table 2 and Figure 1. Oxytocin at baseline was positively related to baseline Y-BOCS scores in the total group (Spearman’s rho = 0.35, n = 36, p = 0.037). The future SRI responders accounted for this correlation (rho = 0.58, n = 16, p = 0.019), that was not retrieved among non-responders (rho = 0.17, n = 19, p = 0.48). On the other hand, baseline oxytocin was unrelated to the future response to treatment, to depression scores (MADRS), sex, age, duration of OCD, history of tics, poor insight, autistic traits and family history of OCD. The age at OCD onset, however, was negatively correlated to baseline oxytocin (rho = -0.36, n = 36, p = 0.030). Median plasma oxytocin of the 12 cases with childhood onset (before 11 years) was higher, 35.4 (31.7, 47.3) pg/ml compared to 21.3 (19.0, 35.6) among the 11 patients with adult onset (from 18 years), MW Z = 2.12, p = 0.034. Baseline plasma oxytocin was also higher among those completing the 12 weeks’ trial, 34.7 (25.8, 40.0) compared to trial discontinuers, 24.2 (20.5, 30.2), MWZ = 2.06, p = 0.039.

**Table 2:** Demographics and clinical characteristics according to baseline plasma oxytocin level

	**All patients n = 36**	**Baseline oxytocin level**		
		**Low mode range: 16.6-24.5 pg/ml n = 13**	**Medium mode range: 27.8-40.3 pg/ml n = 17**	**High mode range: 50.6-67.0 pg/ml n = 6**
Sex, males/females, n (% males)	17/19 (47)	5/8 (38)	10/7 (59)	2/4 (33)
Age, years	40.7 ± 12.7	37.6 ± 12.2	43.5 ± 12.6	39.4 ± 14.7
Cohabiters/singles, n (% cohab.)	20/16 (44)	9/4 (31)	10/7 (41)	1/5 (83)
Any divorce, yes/no, n (% yes)	10/26 (28)	5/8 (38)	5/12 (29)	0/6 (0)
Having children, yes/no, n (% yes)	18/18 (50)	5/8 (38)	10/7 (59)	3/3 (50)
Number of children	1.0 ± 1.2	0.7 ± 0.9	1.2 ± 1.4	1.0 ± 1.1
Full time work, yes/no, n (% yes)	13/22 (37)	4/9 (31)	7/9 (44)	2/4 (33)
OCD, age of onset, years	14.5 ± 6.5	16.2 ± 4.7	13.7 ± 7.6	13.0 ± 6.8
OCD duration, years	26.2 ± 14.8	21.4 ± 14.9	29.8 ± 13.6	26.4 ± 17.1
Familial OCD, yes/no, n (% yes)	14/10 (58)	6/3 (67)	5/6 (45)	3/1 (75)
History of tics, yes/no, n (% yes)	8/26 (24)	2/11 (15)	3/12 (20)	3/3 (50)
Autistic traits, yes/no, n (% yes)	11/23 (32)	5/8 (38)	5/10 (33)	1/5 (17)
History of poor insight, yes/no, n (% yes)	6/27 (18)	1/11 (8)	4/11 (27)	1/5 (17)
Y-BOCS score, baseline	25.3 ± 5.9	23.5 ± 6.2	25.5 ± 5.8	28.8 ± 4.7
NIMH-GOCS score, baseline	9.4 ± 2.0	8.8 ± 2.2	9.6 ± 1.7	10.2 ± 2.1
MADRS score, baseline	12.2 ± 7.8	12.2 ± 8.4	12.2 ± 8.4	12.2 ± 5.9
Previous SRI treatment, yes/no, n (% yes)	12/24 (33)	3/10 (23)	6/11 (35)	3/3 (50)

Mean values ± standard deviations unless otherwise stated. In Chi-2 and one-way ANOVA tests, all the comparisons of these three groups were non-significant. However, for cohabitation the overall result was *χ*^2^ = 4.7 (2), p = 0.09, and comparison between the highest mode and the merged lower modes resulted in *χ*^2^ = 4.4 (1), p = 0.036.

MADRS = Montgomery Åsberg Depression Rating Scale; NIMH-GOCS = National Institute of Mental Health Global Obsessive Compulsive Scale; OCD = obsessive-compulsive disorder; SRI = serotonin reuptake inhibitors; Y-BOCS = Yale-Brown Obsessive Compulsive Scale.

The distinct higher mode of oxytocin (50 – 67 pg/ml) contained 6 individuals, that did not differ from the lower modes, separately or merged (16 – 41 pg/ml) pertaining to the above mentioned clinical variables; however 5 of the 6 were cohabiting, a significantly higher rate compared to the lower modes (*χ*^2^ = 4.41, d.f. = 1, p = 0.036) (Table 2).

### Temporal changes of oxytocin related to SRI treatment

Out of the initial 36 patients, oxytocin samples were obtained from 35 patients after 1 week and from 33 after 4 weeks. Plasma oxytocin medians in the total group after 1 and 4 weeks were 31.2 (22.8, 43.8) and 36.7 (27.0, 43.6), respectively. Median oxytocin plasma levels at the three time points for SRI treated versus placebo treated patients, respectively, were: baseline 32.1 (22.7, 39.5) *vs* 31.2 (22.6, 39.5), after 1 week 30.4 (22.5, 40.5) *vs* 36.1 (25.1, 47.3), after 4 weeks 36.7 (27.3, 43.8) *vs* 37.0 (26.1, 43.6), and intra-individual range 11.6 (5.1, 16.8) *vs* 7.4 (5.8, 12.4). None of these measures or the differences between time point measures differed significantly between treatment groups. Also, when the clomipramine group and the paroxetine group were analyzed separately, no significant differences between them or between them and the placebo group were detected on the different oxytocin measures. According to plasma drug levels at week 4, all SRI patients but one non-responder seemed to comply with treatment.

### Temporal changes of oxytocin related to anti-obsessive response

While the baseline and week 1 samples did not differ between responders and non-responders, the 17 treatment responders (including 1 placebo responder) had higher oxytocin at week 4, than the 16 non-responders (MW Z = 2.31, p = 0.021, missing data = 3). This difference remained if only SRI-treated subjects were included in the analysis (16 responders and 8 non responders) (MW Z = 2.14, p = 0.032, missing data = 3).

The individuals’ changes of plasma oxytocin between the three time points were analyzed and compared between response groups (Table 3). A significant association appeared between final treatment response and the difference of plasma levels between week 1 and week 4, showing an increase of oxytocin among treatment responders and a decrease among non-responders (8.5 (-2.9, 17.5) and -3.1 (-7.5, 2.8), respectively, MW Z = 2.24, p = 0.025). The sole placebo responder had an increase of oxytocin within the highest quartile between baseline and week 4. The intra-individual plasma oxytocin range significantly differentiated responders from non-responders in the total group (median 24.2 (15.7, 37.5) *vs* 8.9 (4.9, 12.7) pg/ml (MW Z = 3.61, p = 0.0003). Furthermore, this oxytocin range also differed between those with autistic traits (n = 11) and those without (5.1 (3.5, 9.7) *vs* 15.6 (7.6, 27.3) MW Z = 2.76, p =0.006, missing data = 2). With Bonferroni adjustment, the two latter findings remained significant.

**Table 3:** Plasma oxytocin in OCD patients: temporal changes according to response category and treatment

	**Total sample**	**SRI responders n = 16**	**SRI Non-responders n = 11**	**Placebo responder n = 1**	**Placebo Non-responders n = 8**	**MW: Z, p**^**a**^	**MW: Z, p**^**b**^	**MW: Z, p**^**c**^
Oxytocin baseline, n = 36	31.3 (22.7, 39.5)	36.0 (25.8, 39.9)	24.5 (19.7, 33.7)	31.2	29.6 (22.3, 39.6)	1.44, 0.15	1.55, 0.12	-0.45, 0.65
Oxytocin week 1, n = 35	31.2 (22.8, 43.8)	30.9 (21.1, 42.6)	30.4 (23.5, 37.4)	-	36.1 (25.1, 47.3)	-0.78, 0.44	-0.47, 0.64	-0.37, 0.71
**Oxytocin week 4, n = 33**	36.7 (27.0, 43.6)	40.1 (30.0, 53.8)	29.4 (23.7, 35.6)	43.6	35.3 (24.1, 42.3)	**2.31, 0.021**	**2.14, 0.032**	-0.68, 0.49
Change from baseline to week 4 (OT-4 – OT-0) n = 33	0.8 (-3.0, 5.6)	1.4 (-6.5, 11.5)	2.4 (-1.3, 4.9)	12.4	-1.9 (-2.7, 2.6)	0.52, 0.60	0.0, 1.0	0.58, 0.56
First week change (OT-1 – OT-0) n = 35	2.8 (-3.8, 5.2)	-5.7 (-13.2, 6.8)	3.7 (2.2, 5.0)	-	2.4 (0.0, 4.8)	-1.36, 0.17	-1.09, 0.28	0.87, 0.39
**Change from week 1 to week 4 (OT-4 – OT-1) n = 32**	1.8 (-6.8, 11.4)	8.5 (-2.9, 17.5)	-0.8 (-5.9, 2.8)	-	-4.7 (-7.5, 3.3)	**2.24, 0.025**	**1.99, 0.047**	0.26, 0.79
**OT range, n = 36**	10.1 (5.5, 16.4)	15.7 (11.8, 25.0)	5.0 (3.7, 9.7)	12.4	6.8 (4.5, 17.5)	**3.61, 0.0003**	**3.38, 0.0007**	0.54, 0.59

Numbers are medians (1^st ^and 3^rd ^quartiles). **Bolded **numbers and texts indicate significant findings. Plasma oxytocin is reported as pg/ml. For response criteria, see Methods.

a: comparison between all responders and all non-responders.

b: comparison between SRI responders and SRI non-responders.

c: comparison between non-responders to SRI and non-responders to placebo.

Only one patient responded to placebo and, hence, is only included in the first (a) statistical analysis. In comparisons between all SRI treated and all placebo treated patients, no significant differences appeared for any of the oxytocin measures.

MW = Mann Whitney statistics; OCD = obsessive-compulsive disorder; OT = plasma oxytocin; OT-0 = baseline sample; OT-1 = week 1 sample; OT-4 = week 4 sample; OT range = intra-individual range between the maximal and the minimal of each patient’s 3 (n = 32) or 2 (n = 4) OT samples; SRI = serotonin reuptake inhibitor.

**Figure 2:**
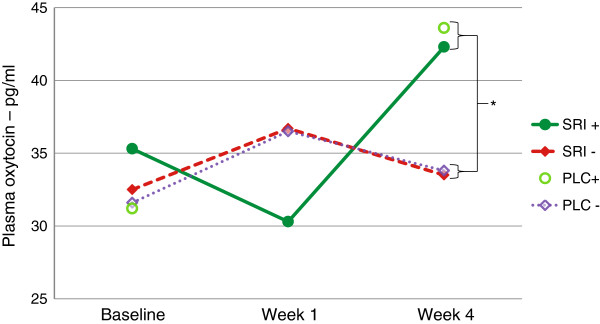
**Plasma oxytocin changes in responders and non-responders to SRI or placebo treatment. ** Mean plasma oxytocin levels (pg/ml) during the first four treatment weeks, among SRI responders (SRI+, n = 16), SRI non-responders (SRI-, n = 11), placebo responder (PLC+, n = 1) and placebo non-responders (PLC-, n = 8), respectively. Mann Whitney statistics were used because of non-normal data distribution. Significant differences were found: at week 4 between all responders and all non-responders (as shown in graph); at week 4 between SRI responders and SRI non-responders; the change from week 1 to week 4 between SRI responders and non-responders (all p < 0.05); for statistical details see Table 3. Missing data: oxytocin at week 1 was missing for the only placebo responder; oxytocin at week 4 was missing in 3 other patients. SRI = serotonin reuptake inhibitor; PLC = placebo. *p < 0.05.

There was a strong negative correlation between both Y-BOCS and NIMH-GOCS% decrease at endpoint and plasma oxytocin change between baseline and first week of SRI treatment; i.e. those with the largest initial increase of plasma oxytocin had the poorest outcome. Reversely, a strong positive correlation was found between oxytocin change between week 1 and 4 and overall improvement in OCD symptoms. None of these measures correlated with improvement of depressive symptoms. The oxytocin range correlated strongly with improvement in OCD and to a lesser extent also with amelioration of depression (Table 4 and Figure 2).

**Table 4:** Correlations of plasma oxytocin measures with anti-obsessive and anti-depressive responses to SRI and OCD severity

	**Y-BOCS, % decrease at endpoint**	**MADRS, % decrease at endpoint**	**Y-BOCS at baseline**	**NIMH-GOCS decrease at endpoint**	**PGE score at endpoint**
OT baseline, n = 27	0.23	-0.05	0.52**	0.25	-0.33
OT week 1, n = 27	-0.34	-0.18	0.25	-0.22	0.04
OT week 4, n = 24	0.31	0.15	0.43*	0.43*	-0.49*
OT change from baseline to week 4, n = 24	-0.14	-0.14	-0.13	-0.20	0.12
OT first week change, n = 27	-0.49**	-0.13	-0.15	-0.40*	0.26
OT change from week 1 to week 4, n = 24	0.58**	0.26	-0.02	0.46*	-0.38
OT range, n = 27	0.59**	0.49**	0.38	0.67***	-0.66***

Only patients treated with SRI are included (n = 27). Nonparametric correlations (Spearman’s rho) are reported. ***Bold-italic*** numbers indicate correlations that remained significant after Bonferroni adjustment, when applied for the first two columns.

MADRS = Montgomery Åsberg Depression Rating Scale; NIMH-GOCS = National Institute of Mental Health Global Obsessive Compulsive Scale; PGE = Patient’s Global Evaluation; OCD = obsessive-compulsive disorder; OT = plasma oxytocin; OT range = intra-individual range between the highest and the lowest of each patient’s 3 (n = 24) or 2 (n = 3) OT samples; SRI = serotonin reuptake inhibitor; Y-BOCS = Yale-Brown Obsessive Compulsive Scale.

*p < 0.05; **p < 0.01; ***p < 0.001.

### Other findings

There were no significant correlations between the previously reported [57] measures of serotonin in whole blood from the same patients and the presently reported oxytocin measures. Specifically, neither the baseline serotonin level nor the decrease of serotonin between baseline and week 1 (that correlated with clinical improvement) showed any connection with the oxytocin measures (all Spearman rho values below 0.10), indicating that these measures are independent.

## Discussion

To our knowledge, this is the first placebo controlled study investigating the effect of SRIs on oxytocin measurements in humans. No difference was found, and the null finding could be due to the limited sample size or other methodological factors. However, the result may be interpreted as reflecting a marked individual variability regarding the reactivity of the oxytocinergic system, due to e.g. genetic factors. In support of this, the individuals with autistic traits had significantly lower intra-individual oxytocin range compared to the others.

On the other hand, we have shown highly significant correlations between dynamic changes of plasma oxytocin during the first four weeks of SRI treatment and subsequent clinical improvement of OCD. This correlation was most pronounced for the range of oxytocin changes: those patients whose oxytocin varied most were also those most improved on all OCD severity measures. Among responders, oxytocin initially decreased and later increased, while the opposite was the case among non-responders (Figure 2). For non-responders, this oxytocin pattern was almost identical for SRI-treated and placebo-treated cases. The one placebo responder seemed to follow the pattern of the SRI responders, with a large elevation of oxytocin levels during treatment, although, unfortunately, the crucial one-week sample was missing in this very case.

Since SRI treatment *per se* did not induce significant changes of plasma oxytocin as compared to placebo, our interpretation is that the findings indicate an association between plasma oxytocin changes and some sequence of events within the brain specifically involved in the anti-obsessive response to SRIs. Specificity for anti-obsessive effects is supported by the considerably lower, mostly insignificant correlations between oxytocin measures and the changes of MADRS scores. The one correlation that emerged may well be the result of depression attenuation, secondary to the OCD improvement. Since plasma oxytocin constitutes only an indirect representation of cerebral events, the nature and direction of these events cannot be resolved from our study. Hypothetically, our data may have resulted from temporal processes (e.g. changes of receptor sensitivities), where the oxytocin system in general changed its activity in opposite directions (due to e.g. genetic polymorphisms) among responders compared to non-responders, the net result being an increased activity. However, in the only relevant previous study [53], CSF-oxytocin increased overall among clomipramine responders, but the best anti-obsessive response was correlated with the least increase or even decrease of CSF-oxytocin. CSF and plasma both constitute imperfect “windows” through which to look at processes in relevant parts of the brain, each summing up different compartment activities. Then, both study results could be explained if an “OCD-crucial” part of the oxytocin system decreased its activity, while other parts non-specifically increased their. In the CSF study, only those with the most significant improvement may have decreased their “OCD-crucial” production sufficiently to prevail over the non-specific increase. In our study, supposedly, the “OCD-crucial” decrease started early on, noticeable after one week, while the non-specific increase lagged after. In any case, our findings may indicate that the oxytocin neurons in responders are more responsive to serotonergic influence than those in non-responders, as indicated by their wider range of oxytocin levels.

Another finding was the replication of a positive relationship between oxytocin levels and Y-BOCS scores in untreated OCD patients, as found by Leckman et al. [41]. In their study, however, this relation was only present in non-tic -related OCD. As seen in Table 2 and Additional file 1: Figure S1, we could not identify any difference of this relation between subtypes. On the other hand, we found a negative association between baseline oxytocin and age at OCD onset, possibly due to a wider range in our study. Since childhood onset OCD may have different etiological factors, e.g. autoimmune mechanisms, these may hypothetically be associated with higher plasma oxytocin. Unfortunately, no data on possible autoimmune OCD was available concerning our patients. A difference between our studies is that Leckman and coworkers measured oxytocin in CSF while the present study was based on plasma levels. In our study, however, the more compelling correlation in future SRI responders and lack of correlation in SRI non-responders may be interpreted as support for a “neuroendocrine subtype” of OCD, where elevated oxytocin may be involved, and which is associated with SRI response.

### Central versus peripheral oxytocin compartments

In the present study it was not possible to identify intra-cerebral events; thus the nature of the implied central nervous counterparts giving rise to and mediating our plasma findings is an unresolved issue. Almost all plasma oxytocin is released from the magnocellular neurons in the SON and PVN via the posterior pituitary. The functional links between the neurohypophyseal release and the intra-cerebral circuits operating with oxytocin are as yet poorly understood; but even if they could be regulated separately [20,23,26], they are most likely to interact. In a study were both CSF and plasma oxytocin levels were analyzed in the same individuals [64], central and peripheral oxytocin measures did not correlate with each other, while both correlated with some measures of suicidality. In various experiments, elevated oxytocin has been linked to relaxed, affiliative situations, implying anxiolytic and antidepressant effects [47,65], but in other experiments oxytocin is increased in relation to stress [26,66]. These disparate findings indicate that different segments of the central oxytocin system may act in different directions. The difficulty of predicting effects within the oxytocinergic system is further underscored by recent studies were intranasal oxytocin induced lowered mood in women with postnatal depression [67], and increased agonistic behaviors with dysregulated HPA axis in piglets [68], respectively, in both cases contrary to expectation.

### Possible influence of the clinical setting and affiliative aspects

In 1992, when the patients in this study were enrolled, OCD was regarded as a rare disorder and many psychiatrists in Sweden were not aware that they had treated any OCD patients in their practice. Therefore, most of the patients were recruited through advertisement in the local paper, and only a handful was clinically referred. The patients were nevertheless ill, and although they were mostly in their early forties, they had a mean duration of OCD of 26 years and one third was defined as depressed. Just about half the sample was able to work full time and the majority lived as singles. Most of these patients had not conveyed their symptoms prior to this study, possibly due to shame. Now, they were assigned a well-informed and devoted psychiatrist that fully understood their symptoms and provided hope for improvement. Such circumstances are often put forward in discussions of problematic placebo response in randomized controlled trials. However, in our study, the rate of placebo response was very low, in accordance with the clomipramine studies of the early days [69]. In spite of this, the social bonding and affiliative aspects of the clinical setting could hypothetically have enhanced oxytocin release and thus influenced the results, resulting in the increase of plasma oxytocin that we saw the first week in both placebo treated patients and SRI non-responders. However, in the SRI responders, the decrease of plasma oxytocin at week 1 may correspond to a response-related serotonin-oxytocin interaction that surmounted this affiliative effect. In this perspective, it may also be of interest that the patients that completed 12 weeks of double-blind treatment had higher baseline oxytocin than those that did not. Nonetheless, the substantial increase of plasma oxytocin in our only placebo responder suggests that oxytocin may be relevant for future studies on placebo response. Incidentally, the relevance of our oxytocin measurements for affiliative aspects was corroborated by the higher number of married or cohabiting individuals in the group with highest baseline oxytocin.

### Implications for anti-obsessive SRI mechanisms

According to El Mansari and Blier [14], the neurophysiological change, responsible for improvement in SRI treatment of OCD, is that orbitofrontal pre-synaptic 5-HT_1D_ receptors are down-regulated by long term (8 weeks) treatment with SRIs. This will lead to an increased transmission over 5-HT_2_ receptors, eventually leading to decreased activity in the “OCD loop”, consisting of orbitofrontal cortex, the head of the caudate nucleus, a direct and an indirect pathway through the basal ganglia, the thalamus, and back to orbitofrontal cortex. It remains a possibility that our changes of oxytocin plasma levels only represent peripheral reflections of such serotonergic events without any functional importance. However, the baseline correlation with OCD severity in our and a previous study [41] supports the view that the oxytocinergic system is not merely a bystander.

An alternative hypothesis would be that down-regulation of post-synaptic 5-HT_1A_ receptors, which is known to take place in the hypothalamus during SRI treatment [51], will result in decreased oxytocinergic transmission in relevant parts of the forebrain. The credibility of this hypothesis, though, depends on conceivable links between the oxytocinergic system and the above mentioned, well documented OCD neural circuit. Even if no evidence was found for frontal cortex oxytocin receptors in an autoradiography study of humans [70], more recently and with more advanced technology oxytocinergic fibers of medium density were identified in the medial orbital and the frontal association cortices of rats [23]. Moreover, in a recent study, intranasal oxytocin challenge in humans caused an increased activity of the caudate nucleus (an essential part of the OCD neural circuit), and a significant correlation between plasma oxytocin and caudate activity was reported [71]. Also, nucleus accumbens has been implicated in OCD [72] and receives oxytocinergic innervation from the PVN. It has even been shown that oxytocin and serotonin interact closely in the nucleus accumbens, related to social reward [73]. Accordingly, it is not inconceivable that overactive oxytocinergic neurons of hypothalamic origin contribute to OCD severity by increasing striatal and orbitofrontal engagement. In such case, a SRI-induced down-regulation of hypothalamic 5HT_1A_ receptors [51] may modulate this oxytocinergic over-activity, thereby eventually contributing to an anti-obsessive response. Since the magnocellular neurons in the PVN transmit oxytocin both by axons projecting to amygdala and nucleus accumbens and by hormonal release into the peripheral circulation [22,23], changed regulation of their central activity may well be reflected in plasma oxytocin levels. However, there is a lack of detailed knowledge on the regulation of these separate activities, especially concerning the effects of SRIs. Admittedly, it is reasonable that the entire oxytocinergic system change synchronized under the influence of SRI treatment, but the possibility remains that different parts of the system react differently to serotonergic changes. In such case, the already discussed, seemingly contradictory findings of OCD-oxytocin relationships in the present and a previous study [53] may find an explanation. However, until future studies have shed more light on the regional serotonergic regulation of oxytocin transmission, and the effects of psychopharmacological manipulations, this remains conjecture.

On the other hand, increased oxytocin activity has been linked to anxiolytic effects, exerted e.g. in the amygdala [23] or the median raphe nucleus [45], effects that may also be involved in the reduction of OCD symptoms. Then, the oxytocin decrease in responders after 1 week’s treatment could hypothetically be linked to an increase in anxiety. Interestingly, when starting SRI treatment in panic disorder, an initial paradoxical increase of anxiety is commonly observed [74,75], however, this is less commonly reported in OCD treatment. Since specific anxiety ratings were not included in the present study, we do not know whether the initial decreases of oxytocin correspond to increases of anxiety. If this were the case, however, oxytocin deficit may contribute to an explanation of this intriguing phenomenon.

Further relevance for connections between serotonin and oxytocin in the human brain has been demonstrated by mechanistic studies of 3,4-methylenedioxymethamphetamine (MDMA or “Ecstasy”). It has been shown in humans that one of the acute effects of MDMA intake is elevation of plasma oxytocin together with pro-social effects [e.g. [76]. Since MDMA purportedly exerts its effects through the serotonin transporter and SRI pretreatment blocked the oxytocin elevation, the authors suggest a primary role for serotonin in the effects of MDMA on oxytocin release. According to Hunt et al. [77], MDMA-induced increase of oxytocin depends on 5-HT_1A_ transmission and takes place in the SON and PVN. Interestingly, two cases where MDMA use was related to *de novo* onset of OCD have been reported [78], seemingly consistent with our hypothesis.

One further link between OCD psychopharmacology and oxytocin is provided by the effects of antipsychotic drugs. In OCD, resistant to SRI treatment, the best documented treatment option is to add an antipsychotic; both haloperidol and risperidone have strong short-term data, while those of olanzapine and quetiapine are mixed [17]. Conversely, in patients with schizophrenia, obsessive-compulsive symptoms may emerge related to antipsychotic use, the risk seemingly higher with clozapine and olanzapine than with haloperidol and risperidone [79,80]. The effects of these antipsychotics on the oxytocin system have been investigated [81,82], showing most markedly increased release of oxytocin and activation of oxytocinergic neurons by clozapine, closely followed by olanzapine, while the effects of risperidone and haloperidol were much less pronounced or absent. Accordingly, if oxytocin contributes to obsession and compulsion severity, this may explain the differential effects of antipsychotics as SRI augmentation in OCD treatment as well as *de novo* OCD provocation among patients with schizophrenia.

Thus, we suggest that the present study supports the idea that oxytocin is involved in OCD, but based on our data we cannot conclude on the preferred direction of oxytocin changes during OCD treatment. However, gleanings from other research shift the balance in the direction of an increased activity of some part of the central oxytocin system in OCD, as previously proposed [18,19,39], and that this activity is moderated by SRI treatment.

### Limitations

This study includes a small sample of patients and it was carried out in the early nineties. On the other hand, considering how widely spread SSRI medication is today, it would be a challenge to obtain a group of mainly drug naïve, chronically ill patients such as those included in the present study. The small sample size is to some extent compensated by the low placebo response, as is validated by the significantly higher response rate with SRI compared to placebo treatment. Also, since the two active treatments did not differ on any relevant measures, they were merged in order to increase statistical power. Because the placebo group remained problematically small, the comparisons with placebo should be seen as tentative; however, the response categories within the SRI group have a more reasonable size.

Oxytocin in plasma was measured at three time points; however, in four patients it was only measured twice due to patient related factors. All samples were obtained during the first 4 weeks of SRI treatment; it would have been of interest to measure oxytocin also after 12 weeks in order to follow further changes. However, our measurement schedule was based on the presumption that the biochemical changes appearing during the first phase of treatment are decisive for treatment result; furthermore the risk of drop-outs increases with the length of the study. Oxytocin was only measured in plasma; it would have been preferable to also include CSF levels, but most patients with OCD are likely to refuse spinal tap due to extensive worries regarding its consequences. Oxytocin is released in a pulsatile manner and may also vary in relation to the menstrual cycle and use of oral contraceptives. These factors were not accounted for in the present study, but it seems unlikely that the correlations to response would have appeared as a spurious result of this omission. On the other hand, the RIA method we used for oxytocin analysis, including plasma extraction, belongs to the most reliable types of oxytocin analysis [83]. The importance of plasma extraction for the validity of these analyses has recently been further emphasized [84]. Furthermore, a link between our oxytocin measurements and mental functions is to some extent validated by the association between oxytocin levels and cohabitation status, in line with previous research; see e.g. [85,86].

Recent findings suggest specific interactions between genetic polymorphisms of the serotonin transporter and oxytocin receptor genes [46], which would have been interesting to explore in our patient group. However, at the time of our study such genotyping was not available.

The lack of a relationship between depressive symptoms and oxytocin measures may be due to our patients’ low but variable levels of depressive symptoms, thus representing a type II error. Finally, we did not use specific rating scales for tics, and autistic traits were only measured with a global scale, HAGS. Again, at the time of the study, other instruments for assessing ASD in adults with normal intellectual ability were not developed.

## Conclusions

The effect of SRIs on the oxytocinergic system is complex, and more research is needed to disentangle the net effects in different parts of this system.

The baseline correlation of oxytocin levels and OCD severity, as well as the highly significant associations between changes of oxytocin levels and anti-obsessive (but not anti-depressive) treatment response, support the notion that oxytocin is involved in the pathophysiology of OCD and, furthermore, that oxytocin is involved in the anti-obsessive effect of SRI. Indeed, our findings suggest the possible existence of an oxytocin related neuroendocrine subtype of OCD, perhaps associated with childhood onset. But neither whether OCD is related to an increased or decreased oxytocinergic activity, nor what part of the oxytocinergic system that is mainly involved, is resolved by this study. When related to previous work, however, our most parsimonious interpretation posits an overactive segment of the oxytocinergic system that is down-regulated by long-term SRI treatment. Recently available oxytocin receptor agonists and antagonists as well as genotyping for the oxytocin receptor would be of interest for further explorations of the connections between serotonergic and oxytocinergic mechanisms in OCD and related disorders. Also, oxytocin sampling schedules that cover both the pulsatile release and the temporal changes of oxytocin regulation, suggested by the present study, may further elucidate this issue. Taken together, our findings suggest that OCD should be included in translational research on oxytocin involvement in psychiatric disorders.

## Competing interests

Dr. Uvnäs-Moberg owns shares in Peptonic Medical AB, a company that develops oxytocin as a drug for vaginal atrophy. All other authors declare that they have no competing interests.

## Authors’ contributions

MBH and SB conceived and designed the study. SB recruited the participants and assessed the majority of the patients. KUM was responsible for the laboratory analyses. MBH analyzed and interpreted the results and drafted the manuscript. All authors contributed to the writing and revising of the manuscript and read and approved the final manuscript.

## Pre-publication history

The pre-publication history for this paper can be accessed here:

http://www.biomedcentral.com/1471-244X/13/344/prepub

## Supplementary Material

Additional file 1 **Correlation between baseline plasma oxytocin and baseline severity of OCD according to OCD subtypes.** Severity of OCD is indicated by total score on the Y-BOCS. Subtypes of OCD are indicated by symbols (see legend). Spearman’s rho = 0.35, n = 36, p = 0.037. OCD = obsessive-compulsive disorder; Y-BOCS = Yale-Brown Obsessive Compulsive Scale; ASD = autism spectrum disorder. Click here for file
