# Supplementary material for: Plasma oxytocin changes and anti-obsessive response during serotonin reuptake inhibitor treatment: a placebo controlled study
Source: BMC Psychiatry. 2013 Dec 23;13:344. doi: 10.1186/1471-244X-13-344 (PMC3877985; doi:10.1186/1471-244X-13-344)
Supplement: Additional file 1 — Correlation between baseline plasma oxytocin and baseline severity of OCD according to OCD subtypes. Severity of OCD is indicated by total score on the Y-BOCS. Subtypes of OCD are indicated by symbols (see legend). Spearman’s rho = 0.35, n = 36, p = 0.037. OCD = obsessive-compulsive disorder; Y-BOCS = Yale-Brown Obsessive Compulsive Scale; ASD = autism spectrum disorder. [file 1471-244X-13-344-S1.pptx]

## Slide 1
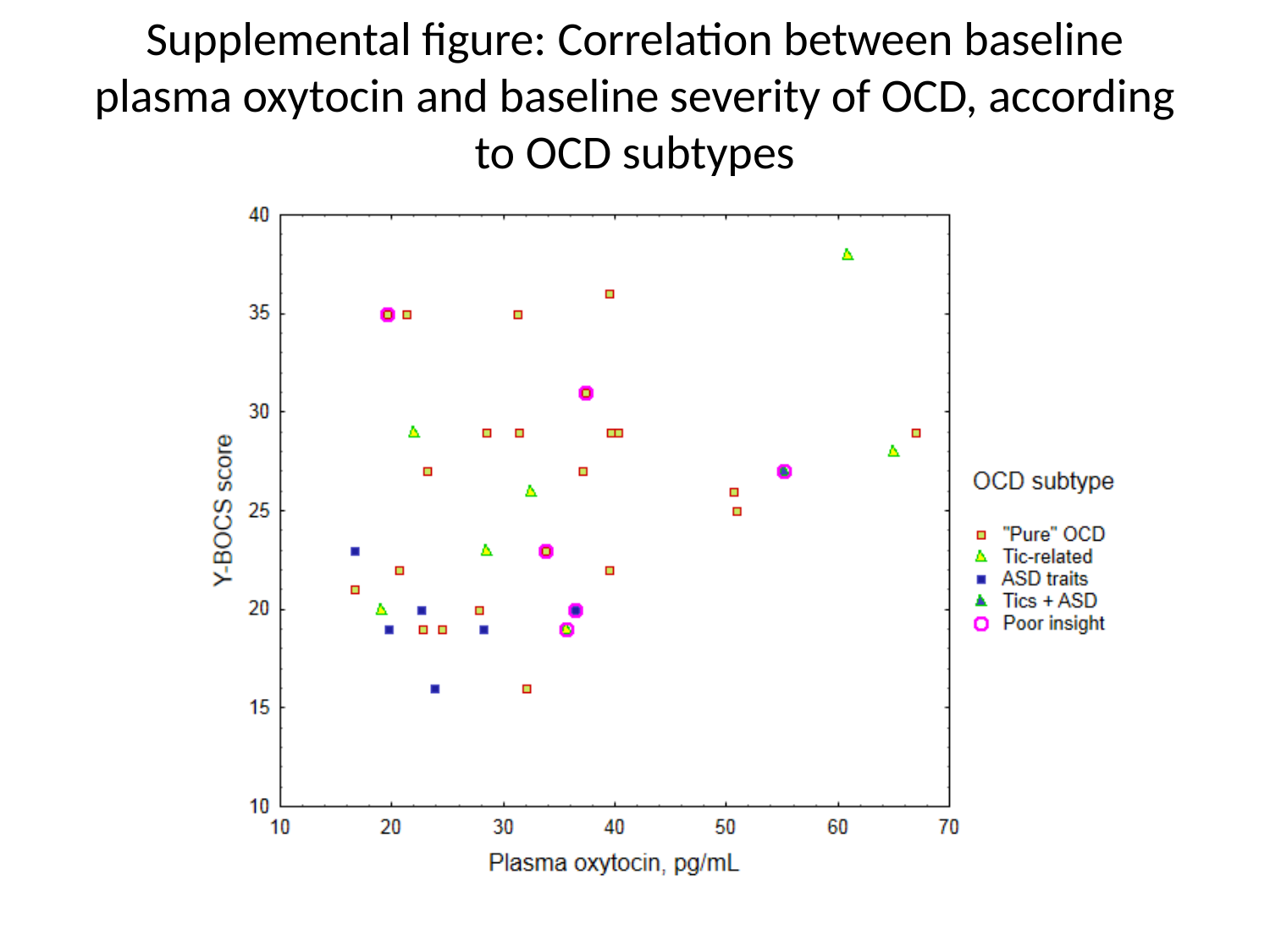

# Supplemental figure: Correlation between baseline plasma oxytocin and baseline severity of OCD, according to OCD subtypes
